# A morphological characterization of the lumbar neural arch in females and males with degenerative spondylolisthesis

**DOI:** 10.1186/s12891-021-04901-6

**Published:** 2021-12-08

**Authors:** Saher Abu-Leil, Asaf Weisman, Yizhar Floman, Fabio Galbusera, Youssef Masharawi

**Affiliations:** 1 The Spinal Research Laboratory, Department of Physical Therapy, The Stanley Steyer School of Health Professions, Sackler Faculty of Medicine, Tel-Aviv University, 69978 Tel Aviv, Ramat Aviv Israel; 2 Israel Spine Center, Assuta Hospital, Tel-Aviv, Israel; 3 Istituto Ortopedico Galeazzi, Milan, Italy

**Keywords:** Spondylolisthesis, Degenerative Spondylolisthesis, Neural arch, Morphometry, Facet arthrosis

## Abstract

**Background:**

Although Degenerative Spondylolisthesis (DS) is a common osseous dysfunction, very few studies have examined the bony morphology of lumbar the neural arch in the population afflicted with DS. Therefore, this study aimed to characterize the neural arch (NA) morphology along the entire lumbar spine in individuals with degenerative spondylolisthesis (DS) and compare them to healthy controls.

**Methods:**

One hundred CTs from a database of 500 lumbar CTs of spondylolisthesis were selected. We excluded vertebral fractures, non-L4-L5 slips, previous surgeries, vertebral spondyloarthropathies, and scoliosis. Scans were divided into a study group of 50 individuals with single-level DS (grades 1–2) at L4–5 (25 males and 25 females), and an age-sex matched control group of 50 individuals. Linear and angular measurements from all lumbar segments included: vertebral canals, intervertebral foramens, pedicles, and articular facets.

**Results:**

Compared with the controls, all individuals with DS had greater pedicle dimensions in the lower lumbar segments (∆ = 1 mm–2.14 mm) and shorter intervertebral foramens in all the lumbar segments (∆range:1.85 mm–3.94 mm). In DS females, the lower lumbar facets were mostly wider (∆ = 1.73–2.86 mm) and more sagittally-oriented (∆10°) than the controls. Greater prevalence of grade-3 facet arthrosis was found only in the DS population (DS = 40–90%,controls = 16.7–66.7%). In DS males, degenerated facets were observed along the entire lumbar spine (L1-S1), whereas, in DS females, the facets were observed mainly in the lower lumbar segments (L4-S1)**.** Individuals with DS have shorter intervertebral foramens and greater pedicle dimensions compared with controls.

**Conclusions:**

Females with DS have wider articular facets, more sagittally-oriented facets, and excessively degenerated facets than the controls. This unique NA shape may further clarify DS’s pathophysiology and explain its greater prevalence in females compared to males.

## Background

Degenerative lumbar spondylolisthesis (DS) is defined as an anterior slip of one vertebra over an adjacent lower vertebra, occurring in a degenerated spinal segment [1–3]. DS is often observed at L4-L5, followed by L3-L4 and L5-S1 [4]. In ~ 66% of the cases, there is a double-level slip [5]. DS prevalence is higher in females (8.4%) than in males (2.7%) [6], sharply increases with age, and is rarely found < 50 years of age [4, 7]. In females, DS is associated with an increased body mass index (BMI) [6] and decreased levels of estrogen production [8]. Radiculopathy with intermittent neurogenic claudication is also associated with DS in aged individuals. However, this condition is not uniquely associated with DS but can also occur in patients afflicted with spinal stenosis, degenerative scoliosis, and segmental instability [9–12].

Some researchers have implied that pathological changes in the spine’s anterior elements, such as degenerative disc disease, are associated with DS’s pathogenesis [13]. Others have focused on changes in the posterior elements [14–17]. Previous studies have indicated that some of the anatomical components of the neural arch (articular facets, pedicles, lamina, spinous process, vertebral canal, and intervertebral foramen) could be related to the pathomechanism of DS [14–17]. Understanding whether the shape of the neural arch is unique in individuals afflicted with DS may help develop specific tools needed to predict either the occurrence of vertebral slippage or its potential for progression.

Although DS is an osseous dysfunction, very few studies have examined the bony morphology of the population afflicted with DS. One study examined the whole lumbar vertebral bodies (VBs) and intervertebral discs (IVDs) with CT scans [13]. Those authors observed that individuals afflicted with DS suffer from generalized degenerative disc disease at all lumbar vertebral levels and are characterized by decreased disc space heights and a kyphotic posture in the upper lumbar segments. Wider pedicles have been observed in various lumbar degenerative diseases [3, 18–21], but only one study directly assessed the neural arch [22]. Goyal et al. compared lumbar vertebral morphology and vertebral dimensions between isthmic spondylolisthesis and DS using MRI and found that the osseous anatomy is significantly different in patients afflicted with DS than those with isthmic spondylolisthesis [22].

### Objectives

Since the neural arch of individuals with DS has been relatively unstudied or compared to healthy populations, it is reasonable to examine possible correlations between VBs and IVD’s unique shape with the neural arch. Our main goal was to analyze the neural arch’s morphometry, including the facets, pedicles, vertebral canals, intervertebral foramens, and compare them with healthy controls. Our second aim was to correlate the neural arch’s morphometry with previously published VBs and IVD data in the same populations [13]. We hypothesized that the shapes of the lumbar articular facets, pedicles, vertebral foramen, and intervertebral foramen along the lumbar spine would correlate with DS.

## Materials and methods

### Human ethics

This study was approved by the Institutional Review board of Tel-Aviv university and the Institutional Review Board of Carmel Hospital, from where the authors obtained the CTs (IRB #2009053). Due to the retrospective nature of the study, both ethics committees waived the need for informed consent. The trial was also prospectively registered in the NIH (#HT5106).

### Study design

Observational, retrospective cross-sectional.

### Study sample

Following ethics approval, 100 lumbar CT scans of individuals aged 50 to 80 were randomly chosen from a hospital database of 500 CTs of DS. The CT scans were equally distributed to two groups: a study group (*N* = 50) and a control group (N = 50). The study group included 50 CT images of individuals with low back pain, symptoms in the lower-limbs, and single-level DS (grades 1–2) at L4–5 (25 males and 25 females). Diagnoses were rendered separately by two senior spine surgeons from Assuta Medical Center Israel based on supine CT scans and standing X-rays images. The control group included CT scans of 50 individuals who were not afflicted with low back pain and DS and were matched according to gender and age (25 males, 25 females, age range 50 to 80) (Table 1). All CT scans with slips other than at L4-L5, evidence of previous surgery, vertebral fractures, spondyloarthropathies, scoliosis, osteoporosis, transitional vertebra, and isthmic spondylolisthesis were excluded [13]. Inclusion criteria for the control group included cases that were examined in the hospital but with no radiological findings and no complaints of back pain.

**Table 1:** Subjects characteristics

Group	Gender	Age (years)	Weight (kg)	Height (cm)	BMI
		(SD)	(SD)	(SD)	(SD)
**Control**	**Male (*n*** **= 25)**	64.7	78.8	174	26.1
		(7.8)	(12.4)	(8.2)	(3.8)
	**Female** **(n = 25)**	61.2	67.6	162.9	25.5
		[7]	(10.3)	(5.4)	(4.1)
**DS**	**Male** **(n = 25)**	65.6	82.2	174	27
		(9.9)	[17]	(7.5)	(4.4)
	**Female** **(n = 25)**	68.6	69.2	159.3	27.2
		(9.3*)	[10]	(3.8*)	(3.7)

*DS* Degenerative spondylolisthesis, *BMI* body mass index; *Significant between the two groups (control/DS) (*p* < 0.05)

### Primary outcome measures

**Fig. 1:**
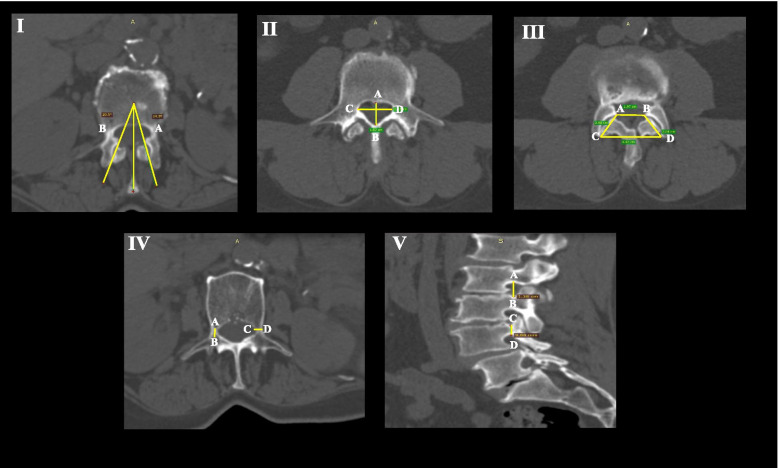
Neural arch measurements. **I)** Transverse facet angle (TFA) = the angle between line A and line C, Transverse inter-facet angle (TIFA) = the angle between line A and line B; **II)** The vertebral canal length (VCL) = the distance between A and B, the vertebral canal width (VCW) = the distance between C and D; **III)** Facet width (FW) = the distance between A and C, Superior inter-facet width (SIFW) = the distance between A and B, Inferior inter-facet widths (IIFW) = the distance between C and D; **IV)** The pedicle length (PL) = the distance between A and B, The pedicle width (PW) = the distance between C and D; **V)** The vertebral foramen height (VFH) = the distance between A and B, The pedicle height (PH) = the distance between C and D

We extracted the following neural arch measurements from all vertebrae from L1-S1 (Fig. 1): ***Foramens:*** superior vertebral canal length and width, and intervertebral foramen height; ***Pedicles:*** pedicle height length and width; ***Articular facets:*** superior and inferior facet widths and inter-facet widths, superior transverse facet angles and superior transverse inter-facet angles and finally, facet arthrosis degree.

### Procedures

All the CT morphological measurements were taken by the same examiner (SA), with 6 years of experience conducting similar measurements, from 2D projections using a computer software program (K-Pacs Workstation Version 1.0.1). We performed Intra-reliability trials on ten CT images before the study’s procedure. The intra-reliability test was conducted by the first author who repeated the exact measurements twice for the same set of 10 CTs. There was a 1-week interval between the two tests.

The degree of facet arthrosis was assessed 6 months after all the morphological measurements were complete. The two examiners (First and second author) were blinded to the diagnosis or identifying features and assessed all 100 scans. Grading was based on the agreement between the two examiners. They evaluated the degree (0–3) and prevalence of facet arthrosis according to Pathria’s classification [23]: 0 = normal facets, 1 = narrowing facets, 2 = narrowing plus sclerosis, 3 = severe osteoarthritis with narrowing sclerosis, and osteophytes. Although Pathria initially intended this classification for x-ray interpretation, it was also used in studies examining CTs and MRIs and showed good reliability [24–26].

### Data analysis

Descriptive statistics were analyzed for all measurements. The Kolmogorov-Smirnov test examined whether the data were normally distributed. A multivariate regression analysis assessed the effects of age, weight, height, and BMI of the measured spinal parameters. Analysis of variances examined the differences between the DS group and controls. The intra-class correlation coefficient (ICC) determined the intra-tester and inter-tester reliability of the measurements. An ICC of > 0.75 was considered good reproducibility, whereas an ICC_3, 1_ < 0.75 was regarded as poor reproducibility [27–29]. We calculated the sample size assuming a 1 mm difference between the means and a difference of 1 mm between the standard deviation as previously observed [13]. For a power of 85% and an alpha of .05, the recommended sample size was 45 subjects for each group. In the case of normally distributed data, the Pearson r correlation coefficients were used to detect any significant correlations between the various morphometrical variables of the neural arch as examined in the current study, and the lumbar VBs and IVDs as previously published in the same populations using the same methodology [13].

## Results

**Fig. 2:**
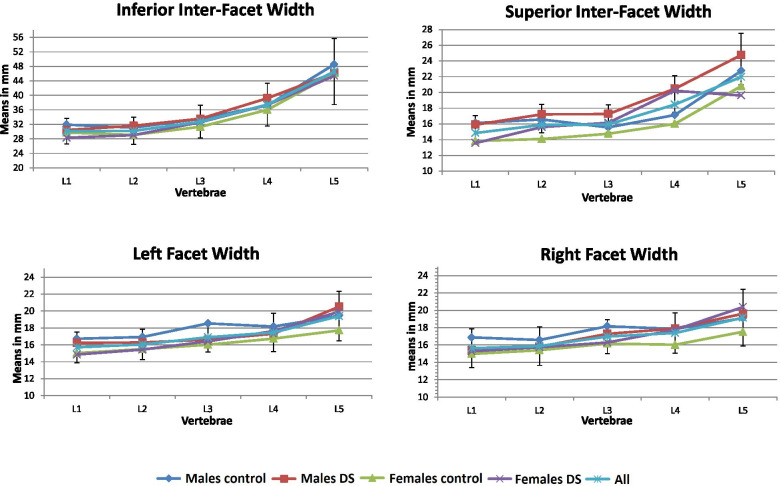
Shape variation of the superior and inferior facet and inter-facet width

**Fig. 3:**
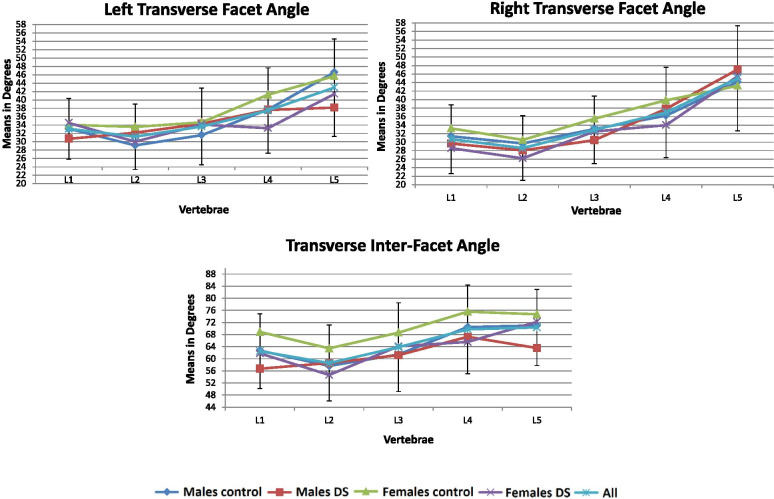
Shape variation of the transverse facet and inter-facet angle

We analyzed a total of 9500 measurements from 100 lumbar CTs (19 measurements in each vertebral level × 5 vertebral levels × 100 CTs). All *p*-values for the Kolmogorov-Smirnov test were > 0.05, indicating a normal distribution of all variables (0.11 < *p* < 0.999). Both intra-tester and inter-tester reliability for all measurements were good (0.85 < ICC _3, 1_ < 1 and 0.85 < ICC_3, 1_ < 0.92, respectively). Males were significantly taller and heavier than the females in both the DS and control groups (*p* < 0.05). Multivariate regression analysis revealed that age, height, weight, and BMI did not affect all measured spinal parameters (0.53 < *p* < 0.994) (Table 1). Patterns of shape variation of all measured parameters along the lumbar spine are described below and illustrated in Figs. 2, 3, 5, 6.

### Articular facets (Table 2, Figs. 2, 3, 4)

The right and left facet width increased distally along the lumbar spine (ΔL1-L5 = 4 mm), the superior inter-facet width increased from L1 to L2 (ΔL1-L2 = 1 mm), remaining unchanged from L2 to L3 and increased towards L5 (ΔL1-L5 = 7 mm). The inferior inter-facet width remained unchanged from L1 to L2 and increasing sharply towards L5 (ΔL1-L5 = 16 mm). The left and right transverse facet angles decreased from L1 to L2 (~ 2°) and increased from L2 to L5 (ΔL2-L5 = 12°-17°).

**Table 2:** Lumbar facet measurements in control and degenerative spondylolisthesis groups

Facet measurements	***Gender***	Group	L1 Mean in mm(SD)	L2 Mean in mm(SD)	L3 Mean in mm(SD)	L4 Mean in mm(SD)	L5 Mean in mm(SD)
**Left Width**	Males	Control	**16.72** **(2.21)**	**16.93** **(1.62)**	^b^**18.45** **(1.89)**	18.16 (2.32)	19.45 (2.69)
		DS	**16.23** **(1.98)**	**16.28** **(1.20)**	^b^**16.62** **(1.47)**	17.31 (1.61)	20.51 (2.42)
	Females	Control	**15.03** **(1.86)**	**15.50** **(2.40)**	**16.02** **(1.55)**	16.73 (1.81)	^a^17.71 (3.23)
		DS	**14.85** **(1.24)**	**15.44** **(1.93)**	**16.41** **(2.04)**	17.63 (3.26)	^a^19.92 (3.45)
**Right Width**	Males	Control	**16.88** **(2.62)**	16.58 (2.24)	**18.16** **(1.96)**	17.85 (2.08)	19.09 (2.30)
		DS	**15.43** **(2.2)**	15.80 (1.84)	**17.29** **(2.19)**	17.90 (3.20)	19.61 (2.71)
	Females	Control	**14.98** **(2.26)**	15.41 (2.27)	**16.17** **(2.11)**	^b^16.03 (1.59)	^a^17.52 (3.14)
		DS	**15.27** **(1.84)**	15.74 (2.55)	**16.30** **(1.54)**	^b^17.76 (2.35)	^a^20.37 (4.93)
**Superior Inter-facet Width**	Males	Control	**16.12** **(1.78)**	**16.58** **(2.12)**	^a^15.56 (2.31)	^b^17.13 (2.45)	**22.74** **(4.76)**
		DS	**15.90** **(1.74)**	**17.22** **(2.73)**	^a^17.26 (2.96)	^b^20.47 (4.83)	**24.74** **(6.04)**
	Females	Control	**13.83** **(2.40)**	**14.09** **(2.98)**	^a^14.76 (1.61)	^b^16.01 (2.85)	**20.80** **(5.54)**
		DS	**13.57** **(2.86)**	**15.61** **(2.66)**	^a^16.14 (3.06)	^b^20.22 (4.53)	**19.62** **(5.86)**
**Inferior Inter-facet Width**	Males	Control	**31.92** **(3.79)**	**31.08** **(4.68)**	33.51 (4.74)	37.19 (6.21)	48.56 (7.71)
		DS	**30.44** **(2.56)**	**31.61** **(3.45)**	33.56 (6.10)	39.20 (6.84)	46.20 (12.71)
	Females	Control	**29.75** **(3.65)**	**29.10** **(3.28)**	31.32 (3.50)	36.01 (4.93)	46.05 (8.73)
		DS	**28.31** **(4.23)**	**29.04** **(3.70)**	32.61 (3.91)	37.35 (5.67)	45.41 (7.23)
**Left Transverse facet**	Males	Control	33.10 (7.95)	29.13 (7.94)	31.58 (10.45)	37.70 (9.39)	46.58 (9.10)
		DS	30.71 (6.34)	32.17 (7.94)	34.30 (11.04)	37.61 (11.54)	38.14 (16.02)
	Females	Control	34.05 (6.91)	33.56 (8.94)	34.64 (9.32)	^b^41.25 (9.10)	45.68 (11.15)
		DS	34.49 (7.64)	30.03 (6.45)	34.11 (5.83)	^b^33.25 (10.70)	41.42 (10.31)
**Right Transverse facet**	Males	Control	31.38 (8.36)	29.71 (7.24)	33.08 (8.30)	36.22 (11.48)	44.17 (13.17)
		DS	29.67 (9.66)	28.06 (6.97)	30.47 (8.96)	37.82 (11.35)	47.08 (12.84)
	Females	Control	^a^33.24 (7.97)	30.52 (8.56)	35.50 (6.89)	^a^39.84 (9.22)	43.25 (12.50)
		DS	^a^28.57 (6.30)	26.21 (7.49)	32.50 (7.65)	^a^34.00 (10.42)	45.47 (10.85)
**Transverse Inter-facet**	Males	Control	62.70 (15.80)	57.63 (13.45)	61.42 (15.48)	70.48 (15.47)	71.00 (11.23)
		DS	56.73 (9.57)	58.59 (11.24)	61.17 (17.65)	67.25 (13.03)	63.55 (15.79)
	Females	Control	68.90 (13.22)	^b^63.44 (16.10)	68.64 (13.35)	^a^75.58 (10.86)	74.72 (10.94)
		DS	61.80 (10.73)	^b^54.64 (9.39)	64.12 (12.18)	^a^65.57 (19.49)	72.20 (12.52)

^a^ significant between the two groups (normal / pathological) < 0.05

^b^ significant between the two groups (normal / pathological) < 0.01; Bold = significant within each groups (males / females)

**Fig. 4:**
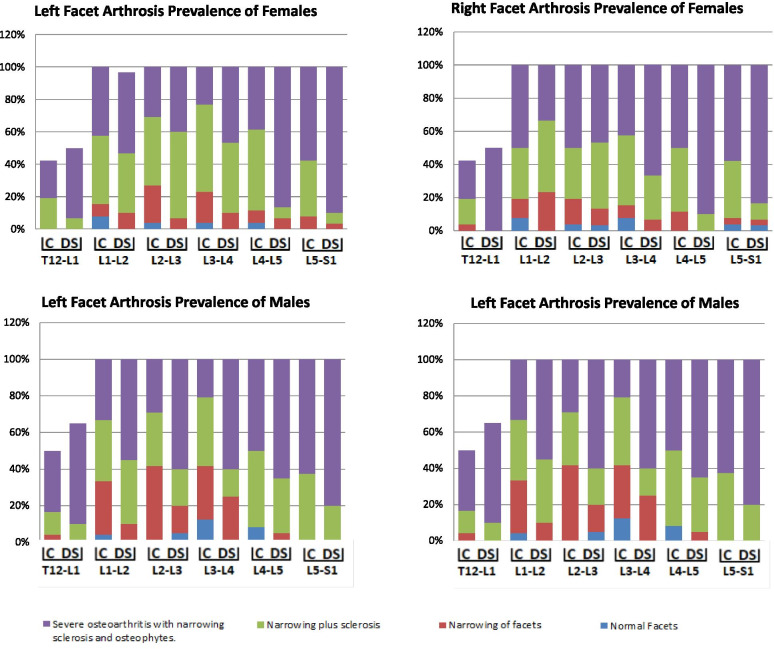
Prevalence (%) of facet arthrosis

Compared with the controls, the left facet was narrower at L3 in males afflicted with DS (∆ = 1.9 mm) and wider at L5 in the afflicted females (∆ = 2.2 mm) (*p* < 0.05). The right facet was wider at L4 and L5 in females afflicted with DS than the controls (∆ = 1.73–2.86 mm). The left facet angle at L4 and inter-facet angles at L2 and L4 were significantly more sagittally-oriented in afflicted females than the controls (∆ = 10°). No significant differences in facet orientation were observed in males in either group.

A greater prevalence of grade-3 facet arthrosis was shown in the DS population compared to the controls (DS = 40–90%, controls = 16.7–66.7%), especially at L5-S1 in both males (80–85%) and females (83.3–90%) and at L4-L5 only in females (86–90%) (Fig. 3). Moreover, a high prevalence of grade-3 facet arthrosis (≥50%) extended along with all lumbar segments in males afflicted with DS, yet, was only concentrated in the lower lumbar segments (L4–5 and L5-S1) in afflicted females.

### Intervertebral foramens and vertebral canals (Table 3 and Fig. 5)

The intervertebral foramen height increased from L1 to L3, remained unchanged at L3-L4, and decreased towards L5-S1 (~ 3 mm). The superior vertebral canal width increased from L1 to L2, remained unchanged at L3, and increased again towards L5 (~ 3 mm). The superior vertebral canal length decreased from L1 towards L3 (~ 2 mm) and remained unchanged at L4, though, increased towards L5.

**Table 3:** Prevalence (%) of facet arthrosis levels of males and females

Lumbar level	Side	Males								Females							
		Control				DS				Control				DS			
**T12-L1**		**0**	**1**	**2**	**3**	**0**	**1**	**2**	**3**	**0**	**1**	**2**	**3**	**0**	**1**	**2**	**3**
	**Right**	0	0	29.2	20.8	0	0	15	50	0	3.8	15.4	23.1	0	0	0	50
	**Left**	0	4.2	12.5	33.3	0	0	10	55	0	0	19.2	23.1	0	0	6.7	43.3
**L1-L2**	**Right**	4.2	16.7	45.8	33.3	5	5	35	55	7.7	11.5	30.8	50	0	23.3	43.3	33.3
	**Left**	4.2	29.2	33.3	33.3	0	10	35	55	7.7	7.7	42.3	42.3	0	10	36.7	50
**L2-L3**	**Right**	4.2	33.3	45.8	16.7	5	5	40	50	3.8	15.4	30.8	50	3.3	10	40	46.7
	**Left**	0	41.7	29.2	29.2	5	15	20	60	3.8	23.1	42.3	30.8	0	6.7	53.3	40
**L3-L4**	**Right**	16.7	29.2	20.8	33.3	0	15	25	60	7.7	7.7	42.3	42.3	0	6.7	26.7	66.7
	**Left**	12.5	29.2	37.5	20.8	0	25	15	60	3.8	19.2	53.8	23.1	0	10	43.3	46.7
**L4-L5**	**Right**	8.3	12.5	20.8	58.3	0	5	50	45	0	11.5	38.5	50	0	0	10	90
	**Left**	8.3	0	41.7	50	0	5	30	65	3.8	7.7	50	38.5	0	6.7	6.7	86.7
**L5-S1**	**Right**	0	0	33.3	66.7	0	0	15	85	3.8	3.8	34.6	57.7	3.3	3.3	10	83.3
	**Left**	0	0	37.5	62.5	0	0	20	80	0	7.7	34.6	57.7	0	3.3	6.7	90

*DS* Degenerative spondylolisthesis; 0 = Normal; 1 = Narrowing of facet joint; 2 = Narrowing plus sclerosis or hypertrophy; 3 = Severe osteoarthritis with narrowing sclerosis and osteophytes

**Fig. 5:**
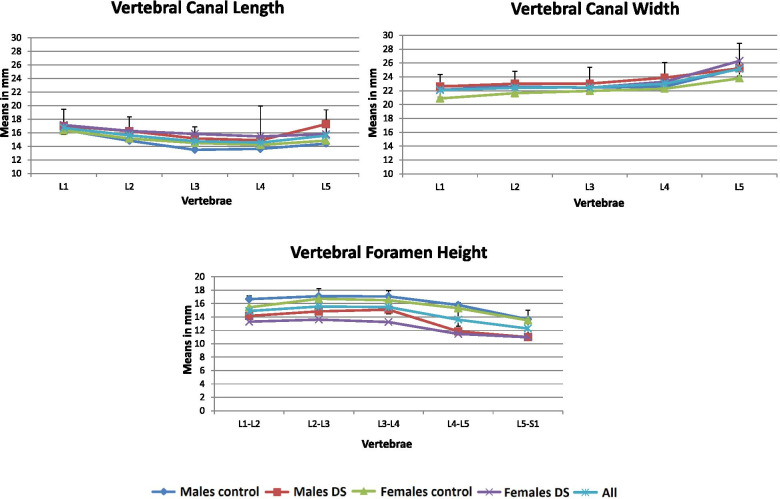
Shape variation of vertebral canal length, width and vertebral foramen height

Compared to the controls, in both males and females in the DS group, the intervertebral foramen was found significantly shorter in all lumbar segments (∆ range: 1.85 mm–3.94 mm). In the DS group, the females’ vertebral canal was wider at L1 (22.15 mm vs. 20.89 mm) and L5 (26.31 mm vs. 23.80 mm) and longer at L3 in females (15.84 mm vs. 14.50 mm) and males (15.17 mm vs. 13.49 mm) and L4 in only females (15.46 mm vs. 14.21 mm) compared to the controls.

### Pedicles (Table 4, Fig. 6)

The left pedicle height decreased from L1 to L2 (1mm), remained unchanged at L3, and decreased again at L4-L5 (~ 3 mm along the lumbar spine). The right pedicle height decreased (~ 2 mm), and the left and right pedicle lengths decreased, both along the lumbar spine (~ 4 mm). The pedicle width increased along the lumbar spine and sharply increased from L3-L5 (~ 6 mm). At L1-L2, the left pedicle width was significantly smaller in afflicted females than the controls (Δ 1.3 mm at L1, Δ 0.8 mm at L2). At L3-L5, the following pedicle measurements were greater in the DS population than in the controls: L3-right lengths in males (Δ 1 mm); L4-left heights in males (Δ 1.87 mm) and females (Δ 1.75 mm), left lengths in males (Δ 1.07 mm) and females (Δ 1.47 mm) and right lengths (Δ 1.37 mm) and width in males (Δ 2.14 mm); L5-left length (Δ 1.26 mm) and height (Δ 1.89 mm) in only females.

**Table 4:** Lumbar vertebral canal measurements in control and degenerative spondylolisthesis (DS) groups

Measurements	Gender	L1 Means in mm (SD)		L2 Means in mm (SD)		L3 Means in mm (SD)		L4 Means in mm (SD)		L5 Means in mm (SD)	
		***Control***	DS	Control	DS	Control	DS	Control	DS	Control	DS
**Canal Length**	M	16.42 (2.91)	**16.98** **(3.17**)	14.81 (2.44)	16.24 (4.06)	**13.49** **(1.61)**	*15.17 **(3.02)**	13.62 (1.75)	14.89 (3.31)	14.39 (2.22)	17.26 (6.79)
	F	16.33 (1.80)	17.14 (3.14)	15.14 (1.64)	16.27 (2.79)	**14.50** **(1.50)**	**15.84 **(2.38)**	14.21 (2.12)	*15.46 (2.36)	14.84 (3.09)	15.79 (3.14)
**Canal Width**	M	**22.68** **(2.67)**	**22.63** **(2.70)**	22.59 (2.35)	23.01 (2.79)	22.47 (2.73)	23.03 (2.86)	22.59 (2.66)	23.89 (3.43)	25.23 (3.69)	25.24 (3.37)
	F	**20.89** **(1.61)**	22.15* **(2.01)**	21.65 (2.28)	22.50 (2.10)	21.97 (2.49)	22.44 (3.47)	22.29 (3.52)	23.30 (2.59)	23.80 (3.45)	*26.31 (4.32)
**Vertebral foramen height**	M	**16.66** **(2.05)**	14.15* **(2.41)**	17.10 (2.72)	*14.83 (2.64)	**17.07** **(2.73)**	*15.08 **(2.26)**	15.78 (3.20)	11.84 (2.39)	13.64 (3.00)	*10.99 (3.41)
	F	**15.14** **(1.80)**	*13.29 **(2.83)**	16.71 (2.23)	*13.61 (3.11)	**16.49** **(2.38)**	*13.24 **(2.34)**	15.30 (2.06)	11.46 (2.66)	13.50 (2.23)	*10.97 (2.34)

(M) = Male, (F) = Female, (*) = significant between the two groups (control /study) respectively with gender (*p* < 0.05); Bold = significant between males and females (*p* < 0.05)

**Fig. 6:**
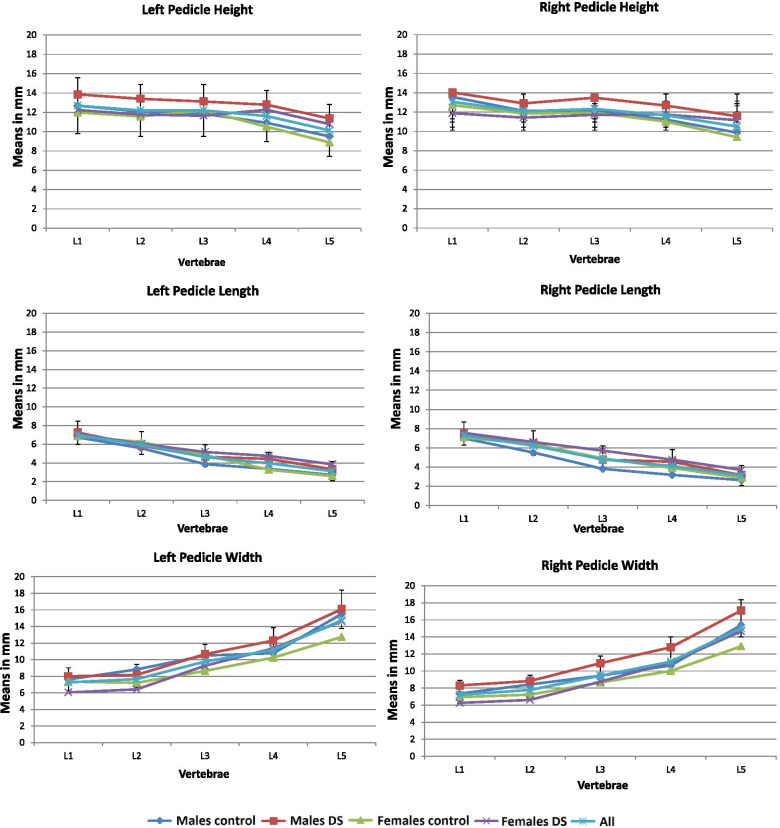
Shape variation of pedicle height, length and width

### Correlations between the neural arch, VBs, and IVDs

We compared the current neural arch’s measurements with previous measurements of the VBs and adjacent IVDs in the same populations for this aim [13]. Only in afflicted females, the greater the lordotic L4 VB wedging and posterior IVD height were, the greater the pedicle dimensions appeared in L4 and L5 (0.41 < Pearson’s r < 0.5; *p* < 0.5) (Table 5).

**Table 5:** Lumbar Pedicle measurements in control and degenerative spondylolisthesis group

Pedicle measurements	***Gender***	Group	L1 Mean in mm(SD)	L2 Mean in mm(SD)	L3 Mean in mm(SD)	L4 Mean in mm(SD)	L5 Mean in mm(SD)
**Left height**	Males	Control	**12.68** **(3.40)**	12.07 (3.29)	11.85 (2.50)	^a^10.91 (3.15)	9.50 (3.11)
		DS	**13.85** **(2.80)**	13.40 (2.25)	13.12 (2.29)	^a^12.79 (2.41)	11.36 (3.06)
	Females	Control	**11.98** **(2.93)**	11.57 (2.72)	12.20 (3.47)	^b^10.51 (2.70)	^b^8.88 (2.41)
		DS.	**12.24** **(2.52)**	11.77 (2.50)	11.64 (2.51)	^b^12.26 (2.38)	^b^10.77 (2.17)
**Right height**	Males	Control	13.56 (2.83)	12.13 (2.62)	**12.16** **(2.41)**	11.23 (3.20)	9.88 (3.22)
		DS	14.03 (2.38)	12.90 (2.76)	**13.49** **(1.99)**	12.68 (2.65)	11.58 (2.47)
	Females	Control	12.72 (2.27)	11.86 (2.73)	**11.93** **(1.97)**	11.02 (2.48)	^b^9.41 (2.59)
		DS	11.89 (2.42)	11.43 (2.52)	**11.72** **(2.19)**	11.74 (2.71)	^b^11.16 (2.41)
**Left Length**	Males	Control	6.74 (1.22)	5.59 (1.23)	**3.88** **(1.25)**	^b^3.37 (0.8)	2.72 (0.98)
		Control	7.26 (1.77)	5.86 (1.43)	**4.66** **(1.45)**	^b^4.44 (1.37)	3.33 (0.88)
	Females	DS	6.87 (1.38)	6.24 (1.48)	**4.85** **(1.14)**	^b^3.28 (1.13)	^b^2.60 (1.00)
		Control	7.10 (1.62)	6.04 (1.56)	**5.18** **(1.47)**	^b^4.75 (1.33)	^b^3.86 (1.21)
**Right Length**	Males	DS.	6.99 (1.41)	5.51 (1.39)	^b^**3.81** **(1.30)**	^b^3.18 (0.79)	2.63 (0.91)
		Control	7.51 (1.30)	6.44 (1.75)	^b^**4.76** **(1.30)**	^b^4.55 (1.47)	3.15 (0.88)
	Females	DS	7.08 (1.33)	6.37 (1.41)	**4.91** **(1.39)**	3.90 (2.68)	2.88 (1.20)
		Control	7.55 (1.62)	6.60 (1.61)	**5.70** **(1.67)**	4.77 (1.76)	3.69 (1.27)
**Left Width**	Males	DS	**7.63** **(2.91)**	**8.83** **(2.48)**	**10.50** **(2.66)**	10.78 (3.96)	**15.51** **(4.56)**
		Control	**8.00** **(1.41)**	**8.17** **(1.91)**	**10.67** **(1.60)**	12.31 (1.90)	**16.09** **(3.12)**
	Females	Control	^b^**7.36** **(1.34)**	^a^**7.21** **(1.29)**	**8.65** **(2.04)**	10.25 (2.59)	**12.74** **(3.84)**
		DS	^b^**6.06** **(1.38)**	^a^**6.42** **(1.38)**	**9.27** **(2.02)**	11.37 (2.24)	**14.62** **(3.09)**
**Right Width**	Males	Control	**7.35** **(2.20)**	**8.43** **(2.20)**	**9.46** **(2.97)**	^a^10.65 (3.48)	**15.38** **(4.46)**
		DS	**8.31** **(1.99)**	**8.84** **(1.59)**	**10.93** **(2.09)**	^a^12.79 (2.50)	**17.10** **(2.84)**
	Females	Control	**6.95** **(1.30)**	**7.23** **(1.58)**	**8.66** **(1.97)**	10.02 (3.09)	**12.90** **(3.87)**
		DS	**6.27** **(1.29)**	**6.62** **(1.55)**	**8.77** **(2.21)**	11.01 (2.56)	**14.67** **(2.27)**
**Transverse Pedicle**	Males	Control	10.25 (3.91)	10.38 (4.79)	10.67 (4.41)	**10.58** **(3.93)**	13.83 (5.28)
		DS	10.85 (2.96)	11.55 (3.99)	13.00 (3.63)	**11.55** **(4.62)**	10.36 (5.77)
	Females	Control	11.46 (3.55)	11.12 (4.11)	12.35 (3.78)	^b^**13.80** **(4.58)**	12.81 (6.94)
		DS	12.14 (4.36)	12.64 (4.94)	12.25 (4.98)	^b^**12.70** **(4.87)**	12.42 (6.48)

*DS* degenerative spondylolisthesis; ^a^ = significant between the two groups (normal / pathological) < 0.05;^b^ = significant between the two groups (normal / pathological) < 0.01; Bold = significant within each groups (males / females)

## Discussion

To the best of our knowledge, this is the first study to compare the entire neural arch’s morphology along the entire lumbar spine in individuals afflicted with DS. This morphological data is very similar to other published data (normal and pathological) [30] from different populations and with varying sample sizes, thus strengthening our methods and conclusions (Table 6) [18, 19, 21, 30–37]. For example, in the mentioned studies, the pedicle width increases and the pedicle height decreases along the lumbar spine (L1-L5). In the current results, the neural arches of all individuals afflicted with DS were characterized by shorter intervertebral foramens in all lumbar segments and greater pedicle dimensions in the lower lumbar spine than the controls’. In females afflicted with DS, the lumbar neural arch was characterized by wider articular facets, more sagittally-oriented facets, and excessively degenerated facets compared with the controls.

**Table 6:** Mean pedicle measurements in the current study compared with previous radiological studies (in control groups)

Study	Population	Sample size (n)	Mean diameter (mm)	L1	L2	L3	L4	L5
Current study	Israeli	50	PW	7.3	7.9	9.3	10.4	14.1
			PH	12.4	12.06	12.03	10.9	9.4
Singh et al. [31]	Indian	302	PW	9	9.5	10.7	11.8	14.3
Abbas et al. [30]	Israeli	180	PW	7	7.3	8.8	10.7	15.2
			PH	14.9	14.1	14	13	11.7
Mohanty et al. [21]	Indian	102	PW	7.2	7.6	8.4	10.1	13
Marasini et al. [32]	Nepalese	246	PW	7.2	7.6	9.5	10.6	11.3
			PH	15	15.3	15.2	13.5	12.6
Acharya et al. [33]	Indian	50	PW	7.2	7.6	8.9	11.1	13.9
Chadha et al. [18]	Indian	20	PW	6.7	7.2	8.4	10.8	13.5
Kadioglu et al. [34]	Eastern Anatolian	29	PW	8.8	9.7	10.3	10.8	14.6
			PH	14.7	14.5	13.6	13.6	13.4
Mitra et al. [35]	Indian	20	PW	7.3	7.5	8.5	9.7	14.5
			PH	16.4	15.6	15.2	15.3	15.2
Cheung et al. [36]	Chinese	134	PW	5.3	6.7	9.5	11.5	14.7
Bernard and Seibert [19]	American	154	PW	–	8.1	8.7	10.9	14.5
Olsewski et al. [37]	American	42	PW	8.2	8.3	10.0	12.6	16.6
			PH	18.2	17.2	16.9	15.6	13.8

*PW* Pedicle width, *PH* Pedicle height

Previous studies have reported a clear correlation between DS and the facet joints’ sagittal orientation [7, 14, 15, 17, 38]. Individuals with an increased sagittal facet orientation at the L4-L5 are 25 times more likely to suffer from DS than individuals with a lesser sagittal facet orientation [38]. However, Hosoe and Ohmori (2008) reported contrasting results showing no clear correlation between the facet joints’ sagittal orientation and DS [39].

The current results indicate that afflicted females’ lumbar spine is characterized by wider facets at L4 and L5 and more sagittally-oriented facets at L2 and L4. This finding is in line with previous data that observed a correlation between sagittally-oriented facets at L4–5 and DS prevalence [15, 17, 38, 40]. When an increased sagittal facet orientation is combined with excessively degenerated facets at L4-L5, as indicated herein, it is reasonable to suggest that this will further facilitate its anterior slippage in females due to altered mechanical forces and displacement of the center of gravity in a lordotic lumbar spine. This explanation is further supported because we found that the lower lumbar facets become wider, probably as a structural adaptation for the increased mechanical stresses. Conversely, in the current results, males’ facet orientation was not correlated with DS; therefore, it may not play a significant role in DS’s pathomechanism in this population. On the one hand, this detail may explain the higher prevalence of DS in women than in men [6]. On the other hand, we found that degenerated facets in afflicted men extended along the entire lumbar spine (L1-S1) compared with females (L4-S1), which may facilitate the anterior slippage of L4 in this population due to altered mechanical forces. Love et al. claimed that the presence of increased sagittal oriented facet joints at L4–5 in subjects afflicted with DS was a consequence of arthritic changes rather than a direct result of DS [41].

Our findings correspond well with previously published data, where the same subjects afflicted with DS exhibited a significantly greater prevalence of osteophytes along the lumbar spine (L1-S1), thinner IVDs, greater kyphotic IVD’s shapes in the upper lumbar segments (L1-L3), less lordotic IVDs at the lower segments (L4-S1) in females, and more lordotic VBs of L5 in males [13]. Accordingly, when combined with kyphotic degenerated IVDs in the upper lumbar spine (L1-L3), degenerated sagittally-oriented articular facets in the lower lordotic segments (L4-L5) may facilitate the anterior slippage of L4 [13]. These mentioned contributing anatomical and mechanical factors could be added to a previously proposed interactive cyclical model explaining DS’s pathomechanism [13].

We also found greater pedicle dimensions (lengths and heights) at the lower lumbar segments in individuals afflicted with DS. Nevertheless, a comparison with previously published data of the same populations found that only in afflicted females, the greater the vertebral lordosis of the slipped L4 vertebra was and the greater the IVD posterior height at L4–5, the greater the pedicle dimensions were at L4–5 [13]. This detail may be a structural adaption and mechanical compensation for the slippage of L4. It may further explain why the prevalence of DS is greater in females than in males [6]. Nevertheless, this “adaptive” explanation should be interpreted cautiously as all CT’s were taken in the supine position (i.e. non weight-bearing position), thus probably lacking correlation with spinopelvic parameters related to posture.

Finally, as the pedicles act as the superior and inferior osseous borders of the intervertebral foramen, their greater dimensions shorten the foramen’s space causing spinal stenosis in DS. Indeed, our results demonstrated that all lumbar intervertebral foramens were shorter in the DS group compared to the controls. Although the DS population’s clinical symptomatology and functional disabilities were beyond this paper’s scope, this anatomical finding most probably contributed to DS’s clinical presentation in females and males. This aspect is supported by the fact that radiculopathy with intermittent neurogenic claudication is a condition commonly associated with DS in elderly individuals [9–12].

## Conclusion

In all individuals afflicted with DS, the neural arch is characterized by shorter intervertebral foramens in all lumbar segments and greater pedicle dimensions in the lower lumbar spine than controls. In females afflicted with DS, the lumbar neural arch is characterized by wider articular facets, more sagittally-oriented facets, and excessively degenerated facets compared with the controls. This unique shape of the neural arch along the lumbar spine could be related to DS’s pathomechanism and may explain the greater prevalence of DS in females than males.

## Data Availability

Data are available from the corresponding author upon request.

## Abbreviations

DS: Degenartive spondylolisthesis
BMI: Body mass index
VBs: Vertebral bodies
IVDs: Intervertebral discs

## References

[1] Kalichman L, Hunter DJ (2008). Diagnosis and conservative management of degenerative lumbar spondylolisthesis. Eur Spine J.

[2] Jacobs WC, Vreeling A, De Kleuver M (2006). Fusion for low-grade adult isthmic spondylolisthesis: a systematic review of the literature. Eur Spine J.

[3] Newman P (1955). Spondylolisthesis, its cause and effect: Hunterian lecture delivered at the Royal College of Surgeons of England on 10th February 1955. Ann R Coll Surg Engl.

[4] Rosenberg N (1975). Degenerative spondylolisthesis. Predisposing factors. J Bone Joint Surg Am.

[5] Iguchi T, Wakami T, Kurihara A, Kasahara K, Yoshiya S, Nishida K (2002). Lumbar multilevel degenerative spondylolisthesis: radiological evaluation and factors related to anterolisthesis and retrolisthesis. Clin Spine Surg.

[6] Jacobsen S, Sonne-Holm S, Rovsing H, Monrad H, Gebuhr P (2007). Degenerative lumbar spondylolisthesis: an epidemiological perspective: the Copenhagen osteoarthritis study. Spine..

[7] Cinotti G, Postacchini F, Fassari F, Urso S (1997). Predisposing factors in degenerative spondylolisthesis. Int Orthop.

[8] Imada K, Matsui H, Tsuji H (1995). Oophorectomy predisposes to degenerative spondylolisthesis. J Bone Joint Surg (Br).

[9] Drury T, Ames SE, Costi K, Beynnon B, Hall J (2009). Degenerative spondylolisthesis in patients with neurogenic claudication effects functional performance and self-reported quality of life. Spine..

[10] Hasegawa K, Shimoda H, Kitahara K, Sasaki K, Homma T (2011). What are the reliable radiological indicators of lumbar segmental instability?. J Bone Joint Surg (Br).

[11] Hasegewa K, Kitahara K, Hara T, Takano K, Shimoda H (2009). Biomechanical evaluation of segmental instability in degenerative lumbar spondylolisthesis. Eur Spine J.

[12] Markwalder T-M (1993). Surgical management of neurogenic claudication in 100 patients with lumbar spinal stenosis due to degenerative spondylolisthesis. Acta Neurochir.

[13] Abu-Leil S, Floman Y, Bronstein Y, Masharawi Y (2016). A morphometric analysis of all lumbar intervertebral discs and vertebral bodies in degenerative spondylolisthesis. Eur Spine J.

[14] Dai L (2001). Orientation and tropism of lumbar facet joints in degenerative spondylolisthesis. Int Orthop.

[15] Grobler LJ, Robertson PA, Novotny JE, Pope MH (1993). Etiology of spondylolisthesis. Assessment of the role played by lumbar facet joint morphology. Spine..

[16] Berlemann U, Jeszenszky DJ, Bühler DW, Harms J (1998). Facet joint remodeling in degenerative spondylolisthesis: an investigation of joint orientation and tropism. Eur Spine J.

[17] Sato K, Wakamatsu E, Yoshizumi A, Watanabe N, Irei O (1989). The configuration of the laminas and facet joints in degenerative spondylolisthesis. A clinicoradiologic study. Spine..

[18] Chadha M, Balain B, Maini L, Dhaon B (2003). Pedicle morphology of the lower thoracic, lumbar, and S1 vertebrae: an Indian perspective. Spine..

[19] Bernard JT, Seibert CE (1992). Pedicle diameter determined by computed tomography. Its relevance to pedicle screw fixation in the lumbar spine. Spine..

[20] Hou S, Hu R, Shi Y (1993). Pedicle morphology of the lower thoracic and lumbar spine in a Chinese population. Spine..

[21] Mohanty SP, Pai Kanhangad M, Bhat SN, Chawla S (2018). Morphometry of the lower thoracic and lumbar pedicles and its relevance in pedicle fixation. Musculoskelet Surg.

[22] Goyal DK, Tarazona DA, Segar A, Sutton R, Motto MA, Divi SN, et al. Lumbar pedicle morphology and vertebral dimensions in isthmic and degenerative Spondylolisthesis—a comparative study. Int J Spine Surg. 2021;15(2):243–50. 10.14444/8009 PMC8059380 33900981

[23] Pathria M, Sartoris D, Resnick D (1987). Osteoarthritis of the facet joints: accuracy of oblique radiographic assessment. Radiology..

[24] Xu C, Ding Z, Xu Y (2014). Comparison of computed tomography and magnetic resonance imaging in the evaluation of facet tropism and facet arthrosis in degenerative cervical spondylolisthesis. Genet Mol Res.

[25] Abbas J, Hamoud K, Peleg S, May H, Masharawi Y, Cohen H (2011). Facet joints Arthrosis in Normal and Stenotic lumbar spines. Spine..

[26] Stieber J, Quirno M, Cunningham M, Errico TJ, Bendo JA (2009). The reliability of computed tomography and magnetic resonance imaging grading of lumbar facet arthropathy in total disc replacement patients. Spine..

[27] Shrout PE, Fleiss JL (1979). Intraclass correlations: uses in assessing rater reliability. Psychol Bull.

[28] Kramer MS, Feinstein AR (1981). Clinical biostatistics: LIV. The biostatistics of concordance. Clin Pharmacol Ther.

[29] Cicchetti DV (1994). Guidelines, criteria, and rules of thumb for evaluating normed and standardized assessment instruments in psychology. Psychol Assess.

[30] Abbas J, Peled N, Hershkovitz I, Hamoud K. Pedicle morphometry variations in individuals with degenerative lumbar spinal stenosis. Biomed Res Int. 2020;2020(Article ID 7125914):6. 10.1155/2020/7125914 PMC7060404 32185215

[31] Singh V, Prasad SN, Neyaz Z, Bhargava N, Yadav U, Srivastav AK, et al. Computed tomographic Morphometry of lumbar spine in Indian population. Indian J Neurotrauma. 2021.

[32] Marasini RP, Gautam P, Sherchan B, Gurung G, KC BR. (2014). A morphometric study of lumbar spine pedicles in Nepalese population. J Coll Med Sci Nepal.

[33] Acharya S, Dorje T, Srivastava A (2010). Lower dorsal and lumbar pedicle morphometry in Indian population: a study of four hundred fifty vertebrae. Spine..

[34] Kadioglu H, Takci E, Levent A, Arik M, Aydin I (2003). Measurements of the lumbar pedicles in the eastern Anatolian population. Surg Radiol Anat.

[35] Mitra SR, Datir SP, Jadhav SO (2002). Morphometric study of the lumbar pedicle in the Indian population as related to pedicular screw fixation. Spine.

[36] Cheung K, Ruan D, Chan F, Fang D (1994). Computed tomographic osteometry of Asian lumbar pedicles. Spine..

[37] Olsewski J, Simmons E, Kallen F, Mendel F, Severin C, Berens D (1990). Morphometry of the lumbar spine: anatomical perspectives related to transpedicular fixation. J Bone Joint Surg Am.

[38] Boden SD, Riew KD, Yamaguchi K, Branch TP, Schellinger D, Wiesel SW (1996). Orientation of the lumbar facet joints: association with degenerative disc disease. J Bone Joint Surg.

[39] Hosoe H, Ohmori K (2008). Degenerative lumbosacral spondylolisthesis: possible factors which predispose the fifth lumbar vertebra to slip. J Bone Joint Surg (Br).

[40] Toyone T, Ozawa T, Kamikawa K, Watanabe A, Matsuki K, Yamashita T (2009). Facet joint orientation difference between cephalad and caudad portions: a possible cause of degenerative spondylolisthesis. Spine..

[41] Love TW, Fagan AB, Fraser RD (1999). Degenerative spondylolisthesis: developmental or acquired?. J Bone Joint Surg (Br).

